# An Appraisal of the Role of Previously Reported Risk Factors in the Age at Menopause Using Mendelian Randomization

**DOI:** 10.3389/fgene.2020.00507

**Published:** 2020-05-29

**Authors:** Xiaohong Ding, Rong Tang, Jinjin Zhu, Minzhi He, Huasong Huang, Zhenlang Lin, Jianghu Zhu

**Affiliations:** ^1^Department of Pediatrics, The Second Affiliated Hospital of Wenzhou Medical University, Wenzhou, China; ^2^The First Clinical Medical School, Wenzhou Medical University, Wenzhou, China; ^3^The Second Clinical Medical School, Wenzhou Medical University, Wenzhou, China

**Keywords:** endocrine, reproduction, menopause, menarche, mendelian randomization

## Abstract

**Objective:**

Menopause at a young age is associated with many health problems in women, including osteoporosis, depressive symptoms, coronary disease, and stroke. Many traditional observational studies have reported some potential risk factors for early menopause but have drawn different conclusions. This inconsistency can be attributed mainly to unmodified confounding factors. Identifying the factors causally associated with age at menopause is important for early intervention in women with abnormal menopause timing, and for improving the quality of life for postmenopausal women. This study aims to appraise whether the previously reported risk factors are causally associated with early age at natural menopause (ANM) susceptibility.

**Methods:**

We used Mendelian randomization, a statistical method wherein genetic variants are used to determine whether an observational association between a risk factor and an outcome is consistent with a causal effect.

**Results:**

Women with earlier age at menarche (β = 0.34, se = 0.16, *p* = 0.035), lower education level (β = 1.19, se = 0.41, *p* = 0.004) and higher body mass index (β = −0.05, se = 0.02, *p* = 0.027) had greater risk for early ANM. The causal link between early age at menarche and early ANM was replicated using ReproGen consortium data (β = 0.23, se = 0.07, *p* = 0.001). However, a current smoking habit, one of previously reported risk factors, was less likely to be correlated causally with early ANM, suggesting that previous observational studies may not have sufficiently adjusted for confounders.

**Conclusion:**

Our results help to identify the risk factors of ANM via a genetics approach and future research into the biological mechanism could further help with targeted prevention for early menopause.

## Introduction

Menopause is normally defined as the cessation of spontaneous menses for 12 months, marking the end of the reproductive phase of life; it includes functional ovarian failure and a change of hormone profiles (Nelson, 2008). Estrogen decline in the postmenopausal period is associated with numerous pathophysiological conditions, including osteoporosis, depressive symptoms, coronary disease, and stroke (Ahlborg et al., 2003; Vivian-Taylor and Hickey, 2014; Muka et al., 2016; Carrasquilla et al., 2017). Thus, early age at natural menopause (ANM), which shortens the period of exposure to estrogen, is an important problem in women’s health and quality of life (Hernandez-Angeles and Castelo-Branco, 2016).

Some studies have reported potential risk factors causing early menopause, including early age at menarche (AAM), mother’s early ANM, smoking status, nulliparity, and teetotalism (Tawfik et al., 2015; Depmann et al., 2016; Taneri et al., 2016; Mishra et al., 2017; Whitcomb et al., 2018). A recent meta-analysis reported that AAM at 13 years or more can lead to later ANM (Roman Lay et al., 2020). In addition, women who smoke had their menopause more than 1 year prior than never-smoking women without consideration of the cause of menopause (natural or induced) (Oboni et al., 2016). Adiposity is also non-linearly associated with the risk of early natural menopause, but premenopausal hormone therapy use, which could result in menstrual bleeding, may influence its result (Szegda et al., 2017). Besides, mounting evidence suggests that educational level may lead to many diseases, such as coronary heart disease, myopia, and lung cancer (Tillmann et al., 2017; Mountjoy et al., 2018; Zhou et al., 2019). Similarly, a lower educational level was found to be associated with early ANM (Parazzini, 2007; Gold et al., 2013). However, the existence of an association between educational level and ANM seems to be ambiguous according to a systematic review (Canavez et al., 2011). There is limited evidence of a direct relationship between these risk factors and ANM because of a lack of comparability across studies, weak statistical power, and failure to adjust for confounding factors. To minimize the effects of unmeasured confounding factors in distorting the association between early ANM and its suspected risk factors, a more efficient method is required to investigate whether there is a causal relationship.

**FIGURE 1:**
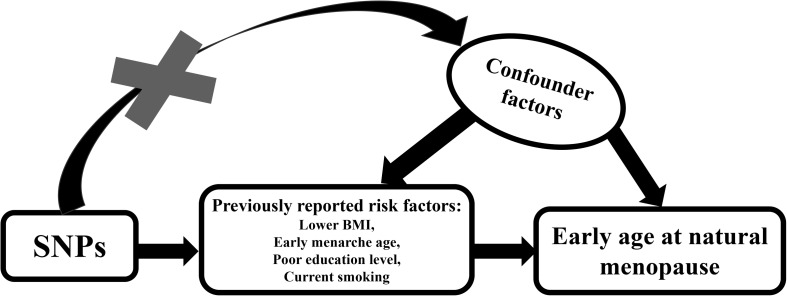
Schematic diagram for Mendelian randomization analysis. Our study aim to estimate the causal relationship between previously reported risks factors and ANM using SNPs as instrumental variables. SNPs should not be associated with any confounding factors.

Mendelian randomization (MR) is an increasingly used statistical method that uses genetic variants to determine whether an observational association between a risk factor and an outcome is consistent with a causal effect (Emdin et al., 2017). It is widely acknowledged that different genotypes determine different intermediate phenotypes. Therefore, if we consider the phenotype an exposure trait, then the association effect between the genotype and a specific disease could be used to assess the role of exposure traits in that disease, under specific basic assumptions that genotype is robustly associated with the exposure, is not associated with confounders and fail to exert influence on the outcome via pathways other than the exposure (Hemani et al., 2018). Because the distribution of genetic variants in a population is random, the function that the exposure traits exert can be distinguished from the effects of confounding factors and may not show the causal association commonly found in traditional epidemiological studies. The schematic diagram for operating a MR is plotted in Figure 1.

In recent years, MR analyses have been widely conducted on single datasets, where data on genotype, risk factor, and outcome were measured for all participants in one specific population (“one-sample Mendelian randomization”). However, advanced analyses on pleiotropy require large sample sizes to guarantee statistical power, which would require data pooling across numerous studies. In reality, such a large program is administratively difficult to organize. As an alternative, summary level data from genome-wide associations study (GWAS) consortia can be used to carry out MR analyses, taking gene exposure measures from one GWAS and gene outcome measures from another GWAS (two-sample MR).

Single-nucleotide polymorphisms (SNPs) is commonly used as a variable in MR analysis (Wu et al., 2010), and the magnitude of the association between SNPs-exposure and SNPs-outcome could be used to estimate the magnitude of the causal effect of the exposure on the outcome (Davies et al., 2018).

To appraise whether the previously reported risk factors are causally associated with early ANM susceptibility, we conducted a two-sample MR analysis using GWAS summary statistics.

## Materials and Methods

### *E*-Value Calculation

The *E*-value was calculated in a website.^1^ The *E-*value can be calculated for an observed risk ratio (denoted RR) by *E*-value = RR + $\sqrt{\mathrm{RR}\times \left(\mathrm{RR}-1\right)}$. If the original risk ratio is below 1, then one first takes the inverse before applying the *E*-value formula (Mathur et al., 2018). We calculated the *E*-value to evaluate the bias from unmeasured confounders in four systematic reviews, which investigated the effect of risk factors (respectively corresponding to AAM, years of schooling, current smoking, body mass index and schooling years) on ANM. A large *E*-value implies that considerable unmeasured confounding would be needed to explain away an effect estimate, whereas a small *E*-value implies little unmeasured confounding would be needed to explain away an effect estimate (VanderWeele and Ding, 2017).

### Data Source and SNPs Selection

Summary statistics for AAM-associated SNPs, body mass index (BMI)–associated SNPs and current tobacco smoking-associated SNPs were extracted from the raw measures in the UK Biobank.^2^ Summary statistics for years of schooling-associated SNPs were extracted from a GWAS study performed by the Social Science Genetic Association Consortium, where 74 loci were identified in 111,349 European individuals (Okbay et al., 2016); the primary data for the study can be downloaded at: http://ssgac.org/Data.php. The effect estimates of these risk factor-associated SNPs on ANM were assessed using the summary statistics from the raw measures in the UK Biobank by Neale Lab, which consists of 111,593 women of European descent.

Furthermore, we extracted summary statistics for AAM-associated SNPs from the GWAS meta-analysis conducted by the ReproGen Consortium (Perry et al., 2014), which consists of 182,416 women of European descent from 57 studies. The effect estimates of these AAM-associated SNPs on ANM were also assessed using the summary statistics from the ReproGen Consortium (Day et al., 2015), which consists of 69,360 women of European descent from 33 studies. Summary statistics from these consortia can be downloaded at the following public website: http://www.reprogen.org/data_download.html.

### Definition of Phenotype

Menarche is defined as the onset of first menstruation in girls. Education attainment is defined as the years spent on school in the SSGAC consortium. Besides, BMI was calculated as weight-to-squared-height ratio (Kg/m^2^). And participants were classified according to whether they were smokers at the time of interview in the UK Biobank.

### SNP Validation and Linkage Disequilibrium Assessment

Maintaining the independence of the selected instruments robustly associated with the exposure is a vital prerequisite for MR analysis, unless measures are taken in the MR analysis to account for any correlations that arise through linkage disequilibrium (LD). An efficient way to ensure that all the instruments are independent is to use clumping against a reference dataset of similar ancestry to the samples in which the GWAS was conducted. Therefore, we performed a clumping procedure (the clumping distance we set is 10,000 kb) in MRBase^3^ or R software with “TwoSampleMR” package^4^ to automatically prune SNPs with linkage dependence. For all pairs of SNPs determined to violate the independence assumption with *r*^2^ > 0.01, we retained only the SNP with the smaller *p*-value.

### Harmonizing Exposure and Outcome SNP Effects

It is imperative to ensure that the effect and standard error for each SNP we identified on the exposure and the outcome corresponds to the same effect alleles. Wrong effect alleles, strand issues, palindromic SNPs, and incompatible alleles are common sources of unexpected bias. Palindromic SNPs can make it difficult to identity the effect allele due to same pair of letters on the forward and reverse strands. Effect allele frequency (eaf) can be used to resolve this ambiguity. If frequency of allele < 0.5 in the exposure study whereas frequency of same allele > 0.5 in the outcome study, two studies have used different reference strands. In this scenario, the outcome alleles should be flipped to match the exposure alleles, and effect alleles are then aligned. However, an eaf may not be a reliable indicator of reference strand when it is close to 0.5 (Hartwig et al., 2016). Palindromic SNPs with intermediate allele frequencies were deleted in case of unexpected bias using “TwoSampleMR” package in R software.

### Pleiotropy Assessment

Another important prerequisite for an MR analysis is that SNPs robustly associated with exposure should not affect the ANM though any horizontal pleiotropic pathways except exposure itself. To evaluate whether there are pleiotropic effects, in which instrumental variables influenced ANM through more than one biological pathway, MR-Egger regression, a widely acknowledged statistical method, was performed to analyze the potential pleiotropism. The pleiotropic effect of the included SNPs is visualized when applying the MR-Egger regression by representing an intercept that deviates from the origin, which may provide evidence for potential pleiotropic effects across the genetic instrumental variables. We also plotted a funnel graph, providing for a visual inspection of symmetry, where any departures may be suggestive of potential directional (or unbalanced) pleiotropy, which indicates whether causal estimates from weaker variants tend to be skewed in one direction (Bowden et al., 2015).

### Heterogeneity Assessment

The minor allele frequency (MAF) differences among different ancestries may result in SNPs that are related not only with the outcome but also the ancestry. To avoid this problem in our MR analysis, we enrolled SNPs and their corresponding summary statistics (*p*-value, beta effect, and standard error) from cohorts that only included European individuals. Nonetheless, other confounding factors such as age, education level and BMI might still exist in the European subgroup, although the two meta-analyses had already modified for potential stratification. Therefore, Cochran’s *Q*-test, calculated as the weighted sum of squared differences between an individual SNP effect and the pooled effect across all SNPs, was performed to assess the heterogeneity among SNPs.

### MR Estimates

We applied the two-sample MR to assess the role of each exposure trait in ANM. Briefly, we selected the SNPs that were strongly associated (*p* < 5 × 10^–8^) with a specific exposure as our instrumental variables and then acquired the corresponding effect estimates from summary statistics. The effect estimates for the selected SNPs on ANM were extracted from the UK Biobank study and the ReproGen Consortium. Next, a two-sample MR analysis was conducted by weighting the effect estimate of each SNP on ANM by its effect on each exposure. These estimates were then pooled using different models to provide a comprehensive summary for the effect of the previously reported risk factors upon ANM.

Previous GWASs stated that the AAM-associated SNPs only account for some of the variance of AAM. If the selected SNPs were weak instruments, MR analysis would therefore be hampered. Therefore, we carried out an robust adjusted profile score, a recently recommended method that considers the measurement error in SNP-exposure effects, is unbiased when there are many (e.g., hundreds of) weak instruments, and is robust to systematic and idiosyncratic pleiotropy.

We used a weighted median approach, which orders the MR estimates from smallest to largest weighted by their inverse variances; then the weighted median estimator is the 50% weighted percentile. According to InSIDE (INstrument Strength Independent of Direct Effect), the weighted median approach generates unbiased estimates of the MR causal effect, provided that more than 50% of the weight comes from valid SNPs. This improves the power of causal effect detection, leads to fewer type I errors than MR-Egger regression, and complements the MR-Egger method to provide more robust effect estimates in MR analysis (Bowden et al., 2016).

### Sensitivity Analysis

Leave-one-out analysis was used to evaluate if the MR estimate was driven or biased by a single SNP that might have a particularly large horizontal pleiotropic effect (Emdin et al., 2017). With this method, we estimated the effect by dropping the included SNPs one by one. Leave-one-out analysis helped to identify SNPs that led to a large change in the causal effect estimate by excluding them sequentially and recalculating the MR estimate.

## Results

### The Risk Factors for Earlier ANM in Traditional Observational Studies

We selected four systematic reviews and meta-analyses including some studies which had adjusted for confounding factors that explored the effect of risk factors (respectively corresponding to AAM, current smoking, BMI, and schooling years) on ANM (Table 1). The results turned out that early AAM, smoking, decreased BMI and lower educational level were associated with early ANM.

**TABLE 1:** The information about four traditional observational studies.

	Study	Population size	Type of adjustment	Main conclusion	*E*-value
Age at menarche	Roman Lay et al., 2020	232010	None	AAM ≥ 13 years was associated with a later ANM: HR = 0.90, 95%CI = 0.84, 0.96	1.36
Current smoking	Zhu et al., 2018	207231	Race/ethnicity/region, education level, and BMI	Never smoker: Reference current smokers: RR = 1.80, 95%CI = 1.66, 1.95	(i)3
Body mass index	Tao et al. (2015)	313482	None	Normal: Reference (i) Underweight: HR = 1.08, 95%CI = 1.03, 1.14 (ii) Overweight: HR = 0.93, 95%Cl = 0.91, 0.96 (iii) Obese: 0.95, 95Cl% = 0.79, 1,15	(i)1.3 (ii)1.28 (iii)1.23
Schooling years	Schoenaker et al., 2014	14620	Smoking, body mass index, physical activity and education	Low: Reference (i) Middle: HR = 0.93, 95%CI = 0.87, 0.98 (ii) High: HR = 0.81, 95%CI = 0.74, 0.89	(i)1.28 (ii)1.58

We also calculated the *E*-value for each review to assess the influence by unadjusted confounding factors. The *E*-values observed were relatively low, suggesting that some unmeasured confounding factors probably played a sufficiently strong role in the causal relationship between risk factors and ANM, especially in non-adjusted models (Table 1).

### SNP Selection and Validation

We obtained 111 SNPs from the UK Biobank study that were robustly associated with AAM (*p* < 5 × 10^–8^). When we assessed the possible causal relation between the length of schooling (years) and early ANM, 16 SNPs were acquired, all of which showed robust association with the length of schooling. Besides, we identified 110 SNPs when investigating the SNP effects on BMI compared to the SNP effects on ANM. These SNPs were robustly associated with BMI. In addition, only 2 SNPs (rs148428140; rs11747772) were qualified in the causal association test between current tobacco smoking status and ANM. Furthermore, we acquired 61 SNPs when exploring the causal association between AAM and ANM using the ReproGen consortium. Besides, all these SNPs were eligible according to the LD independence test.

The MR-Egger regression results and relatively symmetrical appearance of funnel plot revealed that horizontal pleiotropy did not heavily influence the results (Supplementary Figure S1 and Supplementary Table S1). A *Q*-test was also performed to assess the heterogeneity based on its Q parameters. But variability existed broadly in the included BMI-associated and AAM-associated SNPs from the UK Biobank study (Supplementary Table S2).

Information about the characteristics of the included SNPs summary statistics for ANM as well as risk factors is shown in Table 2. The data pertaining to the horizontal pleiotropy analysis and heterogeneity test are presented in Supplementary Tables S1, S2, respectively.

**TABLE 2:** Characteristics of the SNPs summary statistics for ANM and its risk factors.

Trait	First author	Consortium	Sample size	Number of variants	Sex	Population
Age at menarche	Neale	UK Biobank	188644	13791467	Females	European
	Perry JR	ReproGen	182416	2441816	Females	European
Years of schooling	Okbay	SSGAC	182286	7643301	Females	European
Body mass index	Neale	UK Biobank	193570	13791467	Females	European
Current tobacco smoking	Neale	UK Biobank	193956	13791467	Females	European
Age at natural menopause	Neale	UK Biobank	111593	13791467	Females	European
	Day	ReproGen	69360	2418696	Females	European

*ANM, early age at natural menopause.*

### MR Estimates

Based on the UK Biobank study, the standard IVW MR result showed that each additional year in AAM is associated with later ANM (β = 0.34, se = 0.16, *p* = 0.035). Standard IVW showed strong genetic support for a causal association between each additional schooling year and later ANM (β = 1.19, se = 0.41, *p* = 0.004). Standard IVW MR result suggested that there is genetic support for a causal association between higher BMI and earlier ANM (β = −0.05, se = 0.02, *p* = 0.027). On the contrary, standard IVW failed to show genetic support for a causal association between current smoking and later ANM (β = 0.26, se = 1.46, *p* > 0.05). In addition, the association between early age at menarche and early ANM was replicated using ReproGen Consortium data (β = 0.23, se = 0.07, *p* = 0.001).

The results of the different statistical methods for MR analysis evaluating the causal association between the previously reported risk factors and ANM are compiled in Supplementary Table S3 and visualized as a line graph in Supplementary Figure S2. Single SNP analysis is shown in Supplementary Figure S3. The detail of every SNPs we used was provided in Supplementary Data 1–5.

### Sensitivity Analyses

**FIGURE 2:**
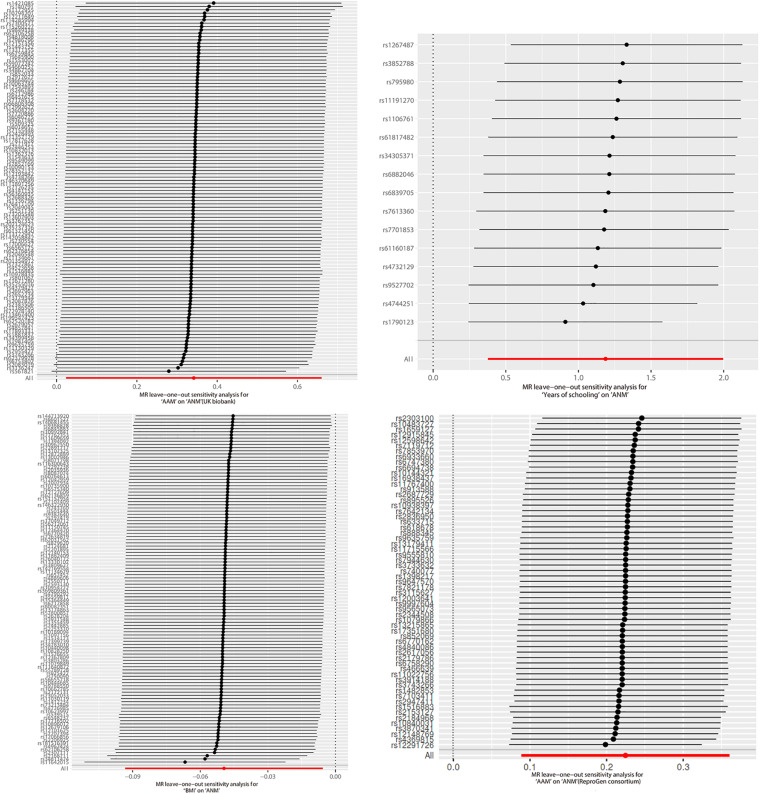
Leave-one-out analysis.

The plot of leave-one-out analysis for each group is shown in Figure 2. Visually, none of the SNPs showed a dramatic bias to the final result. All the primary data of the leave-one-out analysis are reported in Supplementary Data 6–9.

## Discussion

Conducting two-sample MR analysis based on the summary statistics for the previously reported risk factors and ANM of a large European population, we found that each earlier year of AAM, a lower education level and a higher BMI were causally associated with earlier ANM. This result is consistent with a large-scale observational study and casts doubt on other studies that found a negative conclusion, even if they had adjusted for confounders (Mishra et al., 2017). Our study found evidence to support the causal effect of AAM, years of schooling and BMI on ANM by eliminating the potential bias from confounders that exists in traditional observational studies.

Consistent with our results, some researchers revealed that early menarche was significantly associated with the occurrence of menopause (Ozdemir and Col, 2004; Henderson et al., 2008; Nagata et al., 2012). However, Otero and colleges fail to validate the assumption that there was a correlation between AAM and ANM (Otero et al., 2010). A lack of information on duration and regularity of menstrual cycles could influence this result. Short menstrual cycles can increase the frequency of ovulation and deplete the ovarian follicles early, thus leading to early menopause (Cramer et al., 1995). It was reported the interval until regular occurrence of menstrual cycles had a stronger effect on ANM than menarche itself (Nagel et al., 2005). Besides, different results may be attribute to small sample size and different ethnicity.

Age at menarche is an important reproductive parameter associated with some diseases in adulthood (Zacharias and Wurtman, 1969). Late AAM is associated with many disorders in elderly women including cardiovascular disease (Luijken et al., 2017), diabetes mellitus (Karvonen-Gutierrez et al., 2016) and multiple sclerosis (Jiang et al., 2018), while early menarche is related to poor reproductive functioning, including irregular periods (Hillard, 2018), polycystic ovary syndrome (Ibanez et al., 2000) and a slightly increased risk of endometriosis (Nnoaham et al., 2012). At the other end of the reproductive phase, the time of menopause determines the time span of estrogen exposure during a woman’s life, which influences her health (Rogers, 1969). Women with early menopause are more susceptible to climacteric syndrome, a condition with psychological, as well as physiological, aspects that adversely affect their quality of family life (Kaunitz and Manson, 2015). On the other hand, women with late menopause are more likely to have hyperlipidaemia (El Khoudary, 2017), hypertension (Song et al., 2018) and cerebrovascular disease (Raz, 2014). Both AAM and ANM are crucial indicators to predict specific diseases; thus, further understanding of the role of AAM in predicting ANM can help predict some related diseases and help in early intervention.

Although extensive observational studies have reported that the current smoking habit is a risk factor for earlier ANM and for earlier onset of the menopausal transition (Whitcomb et al., 2018), no statistically significant association between the current smoking habit and early ANM was detected in our genetic analysis. A reasonable explanation for the results of previous observational studies is that the risk for early menopause in smokers is dose-dependent and duration-dependent. Some studies reported that there was an increased risk of earlier ANM with both smoking and secondhand smoking (Fleming et al., 2008; Hyland et al., 2016). Concentrations of certain steroid hormones, such as serum estrone, estradiol, and estriol, were lower in active and passive smokers than in non-smokers, suggesting an early decline in ovarian function (Soldin et al., 2011). In a recent, well-organized cohort study, only the subgroup with smoking duration >26 years or smoking ≥10 cigarettes/day showed a significant increased risk for early menopause (Tawfik et al., 2015). The SNPs we included are not grouped according to the duration and dose of smoking, which may attenuate the statistical significance of the pooled-effect estimate.

Epidemiological evidence has shown that lower education level increases the risk of early menopause after adjusting for confounders (Luoto et al., 1994; Gold et al., 2001). The primary data of the current study showed a consistent evidence. The results of observational studies have changed with generations. In studies performed in the 1970s, education level was not associated with menopausal age. However, in more recent studies, a modest association between higher education level and later age at menopause has been reported (Luoto et al., 1994; Kaczmarek, 2007). For example, Dorjgochoo and colleges reported that a higher educational level was associated with later menopause and longer reproductive span. Furthermore, age at enrollment were adjusted to investigate the role of education attainment in ANM (Dorjgochoo et al., 2008). However, some others didn’t find influence of educational level on ANM (Nagel et al., 2005). It is controversial that some studies may be distorted by the consequences of a high education level, such as a high-earning job, wealth, high social status, and awareness of healthy habits. Our genetic study indicated that people with a genetic predisposition toward greater length of schooling showed a significantly lower risk for early menopause.

Lower BMI has also been described as a risk factor for early menopause in observational studies (Morris et al., 2012). Investigators found that higher oestrone production in the adipose tissue of obese women could postpone menopause (Fenton and Panay, 2016). As an endocrine and paracrine organ, adipose tissue is expected to produce substantial adipokines like leptin (Fasshauer and Bluher, 2015), contributing to the regulation of the hypothalamus-pituitary-ovary axis and communicating information about the body’s energy reserve to the brain (Zhang et al., 2015). In contrast, higher risk of early menopause was observed among women with BMI ≥ 35 kg/m^2^ at 18 years, but it was attenuated and no longer significant after adjustment for reproductive factors (Szegda et al., 2017). Moreover, a different meta-analysis indicated that the results changed markedly in the comparison between obese and normal-weight women only in studies that controlled for smoking (Tao et al., 2015). Our genetic analysis supported a causal association between a higher BMI and a higher risk for early menopause, suggesting that adipose tissue of obese women may lead to early menopause via another pathway.

However, all conclusions in this MR analysis are based on the assumption that a *p*-value < 0.05 indicates that an event will not happen. However, numerous researchers and statisticians expressed doubt regarding this assumption (Amrhein et al., 2019). Some researchers have recommended that authors disclose their *p*-values and corresponding sample sizes in published articles rather than just declare that the *p*-value is less or more than 0.05, because the *p*-value reflects the possibility that an event will happen in an adequately large sample size (Laber and Shedden, 2017). In our study, although the MR analysis failed to confirm a causal association between the current smoking habit and earlier ANM because the *p*-value is > 0.05 in the standard IVW model, further investigation will be needed to prove it.

This MR analysis has some strengths. First, the Mendelian law states that alleles are randomly distributed when gametes are formed at meiosis; thus, the causal effect of intermediate phenotype on disease will not be distorted by the confounding factor in MR studies, a problem difficult to avoid in observational studies. Second, this MR analysis adjusted for ethnic factors by selecting SNPs from the summary statistics of women all of the same race. In addition, all datasets we obtained for risk factors deliberately contained only women, which avoided attenuating the association signal between ANM and its risk factors. This rendered our results more accurate and reasonable. Finally, this MR analysis enrolled sufficient samples to improve the reliability of the results. This is the first study to systematically investigate the causal association between the timing of menopause and its potential risk factors using Mendelian randomization.

There are also some limitations of our study. First, this MR analysis only included European individuals, whose data are not globally representative. Second, we did not further investigate the mechanism by which the exposure traits influence the reproductive system. Third, some other risk factors, such as parity, are not available in large GWASs, which prevents the collection of enough information for MR analysis. Thus, we were unable to investigate the relationship between those factors and the risk of early ANM. Finally, although our study suggested that BMI was causally associated with early ANM, it is not absolute due to the heterogeneity of data.

## Conclusion

In conclusion, our results confirmed that women with earlier AAM, a lower educational level, and a higher BMI are at a greater risk from early ANM. However, current smoking habits did not show evidence of causal relation with early ANM, which suggests that previous observational studies may not have sufficiently adjusted for bias. Our results help to identify the risk factors of ANM via a genetics approach. Future research into the biological mechanism could further help with targeted prevention for early menopause.

## Data Availability Statement

The datasets presented in this study can be found in online repositories. The names of the repository/repositories and accession number(s) can be found in the article/Supplementary Material.

## Author Contributions

XD and RT contributed to data collection and manuscript writing. JjZ, MH, HH, and ZL contributed to data processing and figure mapping. JhZ contributed to the study design and data proofread.

## Conflict of Interest

The authors declare that the research was conducted in the absence of any commercial or financial relationships that could be construed as a potential conflict of interest.

## Footnotes

1 https://www.evalue-calculator.com/

2 http://www.nealelab.is/uk-biobank

3 http://app.mrbase.org

4 https://github.com/MRCIEU/TwoSampleMR

## Supplementary Material

FIGURE S1 Funnel plot visualizing the horizontal pleiotropy of the SNPs robustly associated with each risk factor of ANM. Symmetrical funnel plot suggests little horizontal pleiotropy was involved in analysis. **(a)** AAM (UK Biobank), **(b)** schooling years, **(c)** BMI, **(d)** current tobacco smoking, and **(e)** AAM (ReproGen consortium).

FIGURE S2 Comparison of different statistical methods for MR analysis evaluating the causal association between previously reported risk factors and ANM. **(a)** Earlier AAM has a causally positive correlation with earlier ANM according to the analysis conducted using UK Biobank recourse. **(b)** Schooling years has a relationship with earlier ANM. **(c)** correlation was found between higher BMI and early ANM. **(d)** No correlation was found between current tobacco smoking and ANM. **(e)** Association between early AAM and early ANM was found using ReproGen consortium.

FIGURE S3 Single SNP analysis.

## Abbreviations

AAM: age at menarche
ANM: age at natural menopause
BMI: body mass index
eaf: effect allele frequency
GWAS: genome-wide associations study
IVW: inverse variance weighted
LD: linkage disequilibrium
MR.RAPS: robust adjusted profile score
MR: Mendelian randomization
SE: standard error
SNP: single-nucleotide polymorphism.
