# Supplementary figures and images for: An Appraisal of the Role of Previously Reported Risk Factors in the Age at Menopause Using Mendelian Randomization
Source: Front Genet. 2020 May 29;11:507. doi: 10.3389/fgene.2020.00507 (PMC7274172; doi:10.3389/fgene.2020.00507)

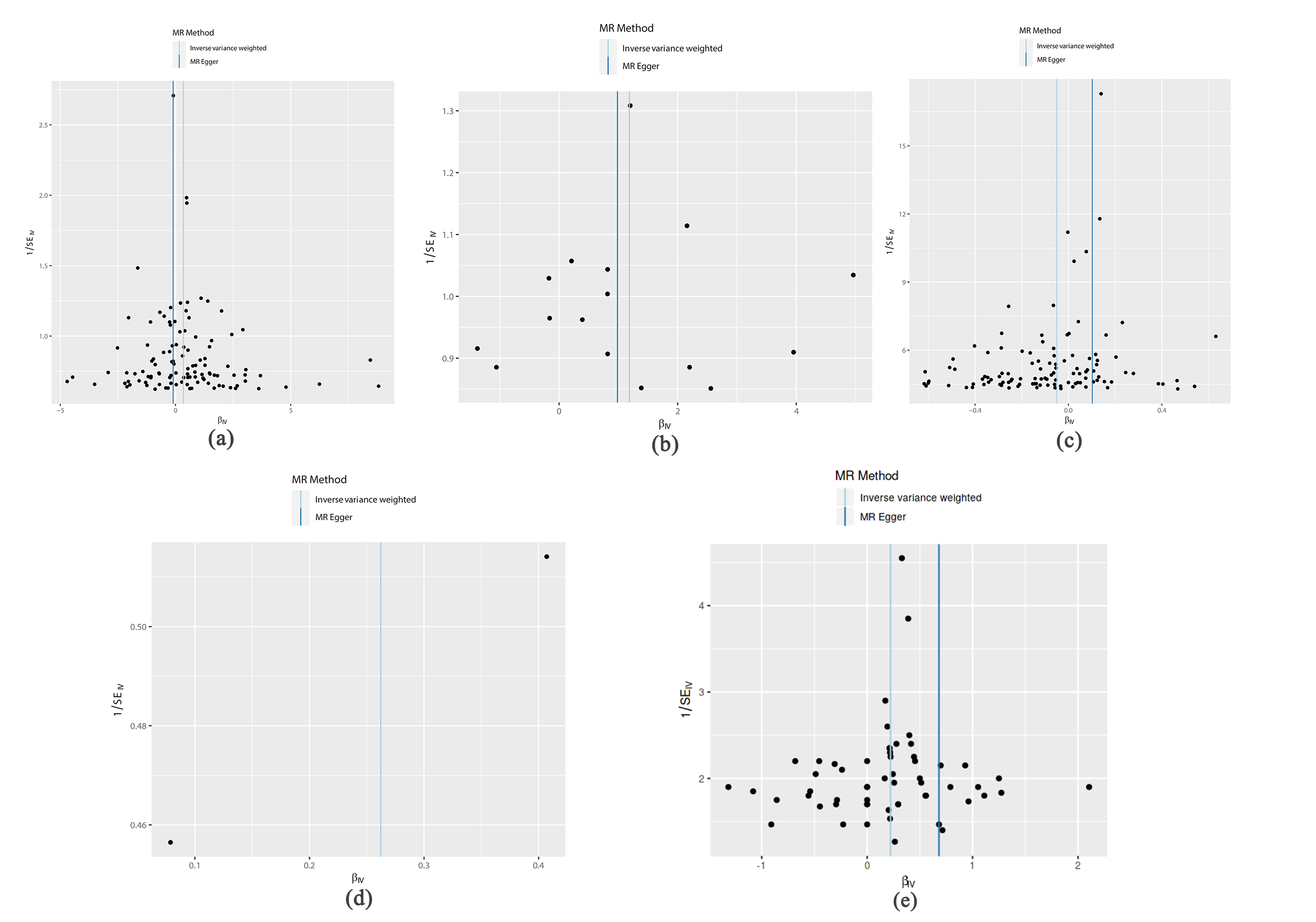

Supplement: FIGURE S1 — Funnel plot visualizing the horizontal pleiotropy of the SNPs robustly associated with each risk factor of ANM. Symmetrical funnel plot suggests little horizontal pleiotropy was involved in analysis. (a) AAM (UK Biobank), (b) schooling years, (c) BMI, (d) current tobacco smoking, and (e) AAM (ReproGen consortium). [file Image_1.TIF]

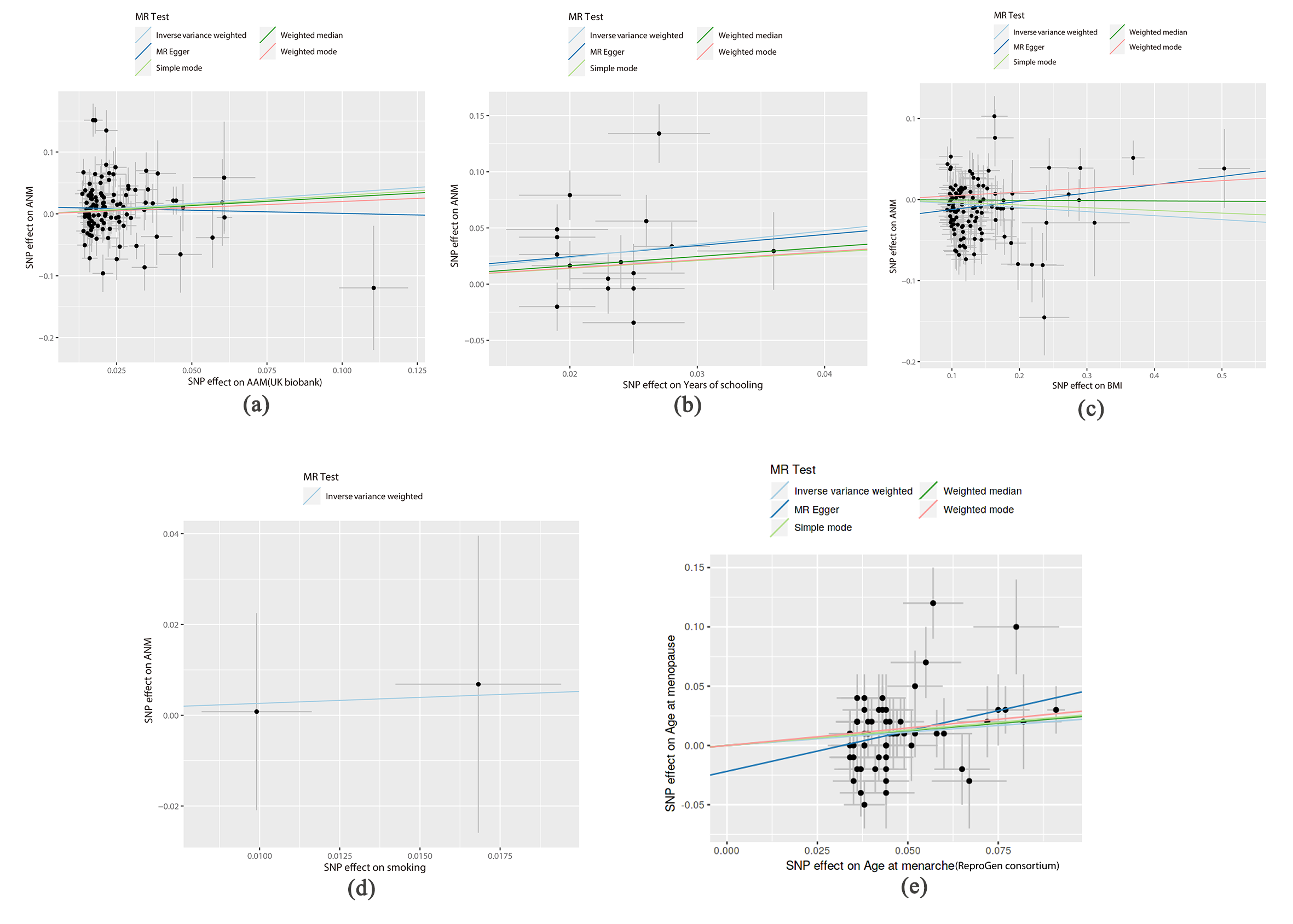

Supplement: FIGURE S2 — Comparison of different statistical methods for MR analysis evaluating the causal association between previously reported risk factors and ANM. (a) Earlier AAM has a causally positive correlation with earlier ANM according to the analysis conducted using UK Biobank recourse. (b) Schooling years has a relationship with earlier ANM. (c) correlation was found between higher BMI and early ANM. (d) No correlation was found between current tobacco smoking and ANM. (e) Association between early AAM and early ANM was found using ReproGen consortium. [file Image_2.TIF]

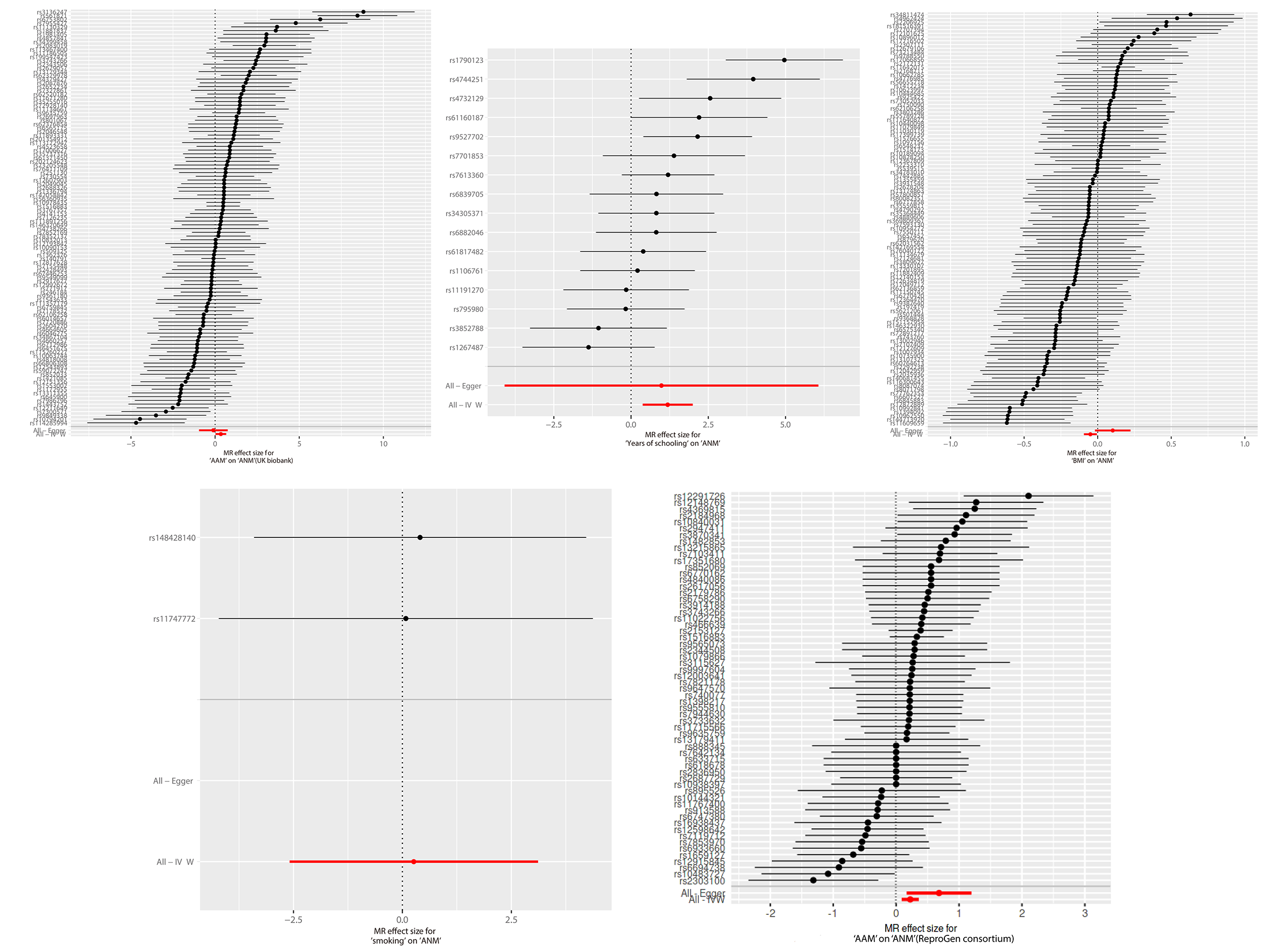

Supplement: FIGURE S3 — Single SNP analysis. [file Image_3.TIF]
