# Supplementary material for: An Appraisal of the Role of Previously Reported Risk Factors in the Age at Menopause Using Mendelian Randomization
Source: Front Genet. 2020 May 29;11:507. doi: 10.3389/fgene.2020.00507 (PMC7274172; doi:10.3389/fgene.2020.00507)
Supplement: Supplementary file 4 [file Table_1.docx]

**Table 1** Summary for directional horizontal pleiotropy tests

| Group | AAM | | | | Schooling years | | BMI | | Current smoking | |
| --- | --- | --- | --- | --- | --- | --- | --- | --- | --- | --- |
|  | UK Biobank | | ReproGen consortium | |  |  |  |  |  |  |
| Egger Regression | Egger intercept | p | Egger intercept | p | Egger intercept | p | Egger intercept | p | Egger intercept | p |
|  | 0.01 | 0.27 | -0.02 | 0.07 | 0.005 | 0.94 | -0.02 | 0.01 | \ | \ |

AAM: early age at menarche; BMI: body mass index
