# Supplementary material for: An Appraisal of the Role of Previously Reported Risk Factors in the Age at Menopause Using Mendelian Randomization
Source: Front Genet. 2020 May 29;11:507. doi: 10.3389/fgene.2020.00507 (PMC7274172; doi:10.3389/fgene.2020.00507)
Supplement: Supplementary file 5 [file Table_2.docx]

**Table 2** Heterogeneity test for each group

| Method |  | AAM | | | |  | Schooling years | |  | BMI | |  | Current smoking | |
| --- | --- | --- | --- | --- | --- | --- | --- | --- | --- | --- | --- | --- | --- | --- |
|  |  | UK Biobank | | ReproGen consortium | |  |  |  |  |  |  |  |  |  |
|  |  | Q | Q_pval | Q | Q_pval |  | Q | Q_pval |  | Q | Q_pval |  | Q | Q_pval |
| MR-Egger |  | 260.2 | 2.20E-14 | 72.2 | 0.12 |  | 40.1 | 2.50E-04 |  | 182.9 | 9.00E-06 |  | \ | \ |
| Inverse variance weighted |  | 263.1 | 1.50E-14 | 76.2 | 0.08 |  | 40.1 | 4.40E-04 |  | 194.7 | 8.70E-07 |  | 0.01 | 0.91 |

AAM: early age at menarche; BMI: body mass index
