# Supplementary material for: An Appraisal of the Role of Previously Reported Risk Factors in the Age at Menopause Using Mendelian Randomization
Source: Front Genet. 2020 May 29;11:507. doi: 10.3389/fgene.2020.00507 (PMC7274172; doi:10.3389/fgene.2020.00507)
Supplement: Supplementary file 6 [file Table_3.docx]

**Table 3** Comparison of the different statistical methods for MR analysis evaluating the causal association between previously reported risk

factors and the timing of ANM

| Methods | AAM | | | | | | Schooling years | | | BMI | | | Current smoking | | |
| --- | --- | --- | --- | --- | --- | --- | --- | --- | --- | --- | --- | --- | --- | --- | --- |
|  | UK Biobank | | | ReproGen consortium | | |  |  |  |  |  |  |  |  |  |
|  | beta | SE | p | beta | SE | p | Beta | SE | p | beta | SE | p | beta | SE | p |
| MR-Egger | -0.1021 | 0.4353 | 0.81491 | 0.6825 | 0.2627 | 0.01184 | 0.9856 | 2.5915 | 0.70942 | 0.1021 | 0.0616 | 0.1004 | \ | \ | \ |
| Weighted median | 0.2699 | 0.1737 | 0.12006 | 0.2493 | 0.08798 | 0.004606 | 0.8189 | 0.3836 | 0.0328 | -0.0041 | 0.0281 | 0.8837 | \ | \ | \ |
| Inverse variance weighted | 0.3417 | 0.1625 | 0.03543 | 0.2247 | 0.06937 | 0.001201 | 1.1868 | 0.4132 | 0.00408 | -0.0494 | 0.0224 | 0.027 | 0.2623 | 1.4545 | 0.85689 |
| Simple mode | 0.2989 | 0.3933 | 0.44887 | 0.2594 | 0.1814 | 0.1579 | 0.6988 | 0.6322 | 0.28642 | -0.0333 | 0.0761 | 0.6629 | \ | \ | \ |
| Weighted mode | 0.2003 | 0.2474 | 0.42003 | 0.2944 | 0.1412 | 0.0413 | 0.7201 | 0.5553 | 0.21432 | 0.0468 | 0.0434 | 0.2832 | \ | \ | \ |

ANM: early age at natural menopause; AAM: early age at menarche
